# Orthostatic Hypotension Is a Predictor of Fatigue in Drug-Naïve Parkinson's Disease

**DOI:** 10.1155/2023/1700893

**Published:** 2023-02-08

**Authors:** Jong Hyeon Ahn, Jin Whan Cho, Jinyoung Youn

**Affiliations:** ^1^Department of Neurology, Samsung Medical Center, Sungkyunkwan University School of Medicine, 81 Irwon-ro, Gangnam-gu, Seoul 06351, Republic of Korea; ^2^Neuroscience Center, Samsung Medical Center, 81 Irwon-ro, Gangnam-gu, Seoul 06351, Republic of Korea

## Abstract

**Introduction:**

Fatigue and orthostatic hypotension (OH) are common and disabling nonmotor symptoms (NMSs) of Parkinson's disease (PD), but none of the studies have reported on the longitudinal association between fatigue and OH.

**Methods:**

Drug-naïve PD patients were recruited from a hospital-based cohort and evaluated with the Parkinson Fatigue Scale (PFS), head-up tilt test, Unified PD Rating Scale, Hoehn and Yahr stage, Montreal Cognitive Assessment, Scale for Outcomes in PD-Autonomic (SCOPA-AUT), Beck Depression Inventory (BDI), Beck Anxiety Inventory, PD Sleep Scale, and medications at the baseline and follow-up visits.

**Results:**

A total of 80 patients were included, and the mean ages were 66.6 and 63.8 years in the fatigue and nonfatigue groups, respectively. The prevalence of fatigue was 17.5% (14/80) at the baseline and follow-up (mean follow-up: 23.3 ± 9.9 months). The prevalence of OH in the fatigue group was 57.1%, and it was significantly higher than that of the nonfatigue group. Six of the 14 patients (42.9%) in the fatigue group had persistent fatigue at the follow-up, and eight of them (57.1%) converted to the nonfatigue group. Logistic regression analysis demonstrated that the changes of BDI and the presence of OH at the baseline were the predictors for fatigue in drug-naïve PD.

**Conclusion:**

Fatigue is a common NMS in PD but can vary depending on the disease course. OH and depression are the most relevant predictors for the development of fatigue in drug-naïve PD. The present study suggests that the management of autonomic symptoms and depression might be helpful for managing fatigue in PD.

## 1. Introduction

Fatigue is a common and disabling nonmotor symptom (NMS) of Parkinson's disease (PD) that affects the quality of life (QoL), even from early stages of the disease [1]. Fatigue is generally defined as an overwhelming sense of tiredness, lack of energy, or need for increased effort [2]. The prevalence of fatigue has been reported from 30% to 58% but varies across studies and the disease stage [1]. Previous cross-sectional studies reported that a wide range of factors are associated with fatigue in PD. However, the results are inconsistent because they influence each other over the course of disease progression [1–7]. A few longitudinal studies have been tried for a better understanding and to figure out the predictors of fatigue [4–9]. They demonstrated that age, female gender, antiparkinsonian medication, excessive daytime somnolence, and emotional apathy were associated with the progression of fatigue [4–9]. Autonomic dysfunction, a common nonmotor symptom of PD, has been reported as an associated factor with fatigue in PD in many cross-sectional studies and suggested as the possible hypothesis for the pathophysiology of fatigue in PD [2, 3, 10, 11]. Among the various autonomic dysfunction, orthostatic hypotension (OH) has been found as the important factor for fatigue in drug-naïve PD [3, 10, 11]. However, the longitudinal effect of OH on fatigue has not been investigated yet. Considering the pathophysiology of fatigue in PD is largely unknown and their treatment is still unsatisfactory, further investigation of predicative factors is needed [12]. In the present study, we investigated longitudinal changes in fatigue and the predictive value of OH in drug-naïve PD.

## 2. Methods

### 2.1. Participants

**Figure 1:**
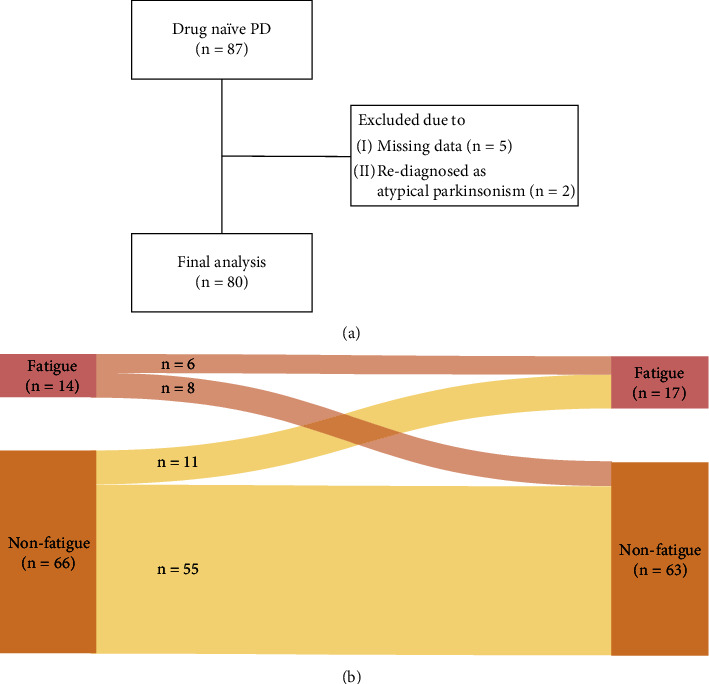
Study overview. A total of 87 patients were screened, and 7 were excluded from the analysis due to missing data and diagnosed as atypical parkinsonism during the follow-up period (a). Six of the 14 (42.9%) patients of the fatigue group had persistent fatigue at the follow-up investigation; in contrast, with medication, eight of them (57.1%) converted to the nonfatigue group. Among the nonfatigue group at the follow-up, eleven patients (16.7%) converted to the fatigue group and 55 (83.3%) remained in the nonfatigue group (b). PD: Parkinson's disease.

We recruited eligible drug-naïve patients with early PD at the Movement Disorders Cohort of Samsung Medical Center. PD was diagnosed based on the United Kingdom Parkinson's Disease Brain Bank Criteria [13], and patients that met the following inclusion criteria were included: (1) less than 36 months from the onset of the motor symptoms of PD at the baseline visit, (2) follow-up for at least 12 months from the baseline investigation, (3) a modified Hoehn and Yahr (H&Y) stage <3 at the baseline visit, and (4) decreased DAT uptake in striatal dopaminergic depletion using 18F-radiolabeled N-(3-fluoropropyl)-2*β*-carboxymethoxy-3*β*-(4-iodophenyl) nortropane PET. We excluded patients with any of the following features: (1) diagnosed with atypical parkinsonism; (2) cardiovascular disease, peripheral neuropathy, diabetic, or other neurological disorders that can cause autonomic dysfunction or fatigue; (3) history of relevant head injury or cerebrovascular diseases, major medical diseases, or musculoskeletal disease; and (4) dementia based on the Diagnostic and Statistical Manual of Mental Disorders (DSM)-5 criteria [14]. A total of 87 patients with drug-naïve PD were included at the baseline, and seven of them were excluded due to insufficient data at the follow-up visit or rediagnosed as not having PD during the study period (Figure 1(a)). This study was approved by the Institutional Review Board (IRB) of Samsung Medical Center, and all subjects provided written informed consent.

### 2.2. Clinical Assessment

The age, sex, disease duration, and follow-up duration were collected for each patient. The Parkinson Fatigue Scale (PFS) was used to investigate the degree of fatigue for the enrolled patients [15]. We assessed the Unified Parkinson's Disease Rating Scale (UPDRS) III [16], the Hoehn and Yahr (H&Y) stage [17], the Montreal Cognitive Assessment (MoCA) [18], the Beck Depression Inventory (BDI) [19], the Beck Anxiety Inventory (BAI) [20], the Scale for Outcomes in Parkinson's Disease-Autonomic (SCOPA-AUT) [21], and Parkinson's Disease Sleep Scale (PDSS) [22]. The same measurements were assessed at the follow-up visit. All clinical assessments, including the UPDRS III and H&Y stage, were conducted at the baseline before the initiation of antiparkinsonian medications and at the follow-up during an on-state. The levodopa equivalent dose (LEDD) [23] and assessment for the use of antidepressants and/or anxiolytics at the follow-up visit were investigated. We classified the patients into fatigue (mean PFS ≥3.3) and nonfatigue groups (mean PFS <3.3) based on fatigue [15].

### 2.3. Head-Up Tilt Test

Participants underwent the head-up tilt test (HUT) at the baseline visit. The patients discontinued medications which could affect the HUT results at least 24 hours before the test. In addition, the participants were prohibited from smoking and drinking beverages containing caffeine on the day of the test. The electrode and BP cuff were attached to the patient, and BP was continuously recorded using the Finometer 1 (FMS, Amsterdam, the Netherlands). OH was defined as a fall of at least 20 mmHg in systolic BP and/or a 10 mmHg fall in diastolic BP within 3 minutes of HUT. In patients with supine hypertension, a reduction in systolic BP of 30 mm Hg was applied [24].

### 2.4. Statistical Analysis

All the data are presented as the mean and standard deviation (SD). The normality of the data was evaluated using the Shapiro–Wilk test. The demographic and clinical features of the fatigue and nonfatigue groups were compared using Student's *t*-tests, the Mann–Whitney *U* test, the chi-square test, or Fisher's exact test depending on the nature of the variable. We conducted a logistic regression analysis to identify the predictors of fatigue in patients with clinical and demographic factors. We included factors that had a *p* value less than 0.1 in our univariate analysis, such as baseline OH, the SCOPA-AUT, BDI, BAI, and PDSS, as well as factors that have been found to be clinically significant in the previous studies, such as age, sex, disease duration, and follow-up duration. To control for potential variability in the data, we standardized the values of the SCOPA-SUT, BDI, BAI, and PDSS which were calculated by the following formula: [(mean follow-up score–mean baseline score)/baseline score SD]. All tests were two-tailed, and the *α* level was set at *p* < 0.05. Statistical analyses were performed using IBM SPSS for Windows (Version 28.0; IBM Inc., Armonk, NY, USA).

## 3. Results

### 3.1. Demographics and Clinical Features

Demographics and clinical characteristics at the baseline and follow-up visits are presented in Table 1. The mean ages were 66.6 ± 11.5 and 63.8 ± 8.4 years in the fatigue group and the nonfatigue group, respectively. The mean follow-up duration was 23.3 ± 9.9 months. The prevalence of fatigue was 17.5% (*n* *=* 14) at the baseline and 21.3% (*n* = 17) at the follow-up visit. The mean PFS score was 3.6 at the baseline, and it was increased to 3.8 at the follow-up visit in the fatigue group. The UPDRS III and H&Y stages did not show significant differences between the fatigue and nonfatigue groups at both time points. The prevalence of OH in the fatigue group was 57.1% (8/14), and it was significantly higher than that of the nonfatigue group (25.8%, 17/66). At the baseline investigation, the fatigue group had a higher score in the SCOPA-AUT total, as well as gastrointestinal, cardiovascular, and thermoregulatory subdomains. The fatigue group had more symptoms of depression (BDI), anxiety (BAI), and sleep disturbance (PDSS) at the baseline as well. When these patients were assessed again at the follow-up visit, the fatigue group showed a higher score in the SCOPA-AUT total, gastrointestinal, and cardiovascular subdomains. The fatigue group had a higher score in the SCOPA-AUT total and BDI at the follow-up visit as well. There were no differences in the total LEDD, but the fatigue group had a significantly higher mean levodopa dose (260.3 ± 134.9) than the nonfatigue group (158.7 ± 160.8). A monoamine oxidase-B (MAO-B) inhibitor was more frequently prescribed in the nonfatigued patients (50.8%) than the fatigue group (17.6%). Logistic regression analysis showed that OH, SCOPA-AUT, and BDI were the associated factors with baseline fatigue (supplementary table 1).

### 3.2. Longitudinal Change of Fatigue

Six of the 14 patients (42.9%) in the fatigue group had persistent fatigue at the follow-up investigation; in contrast, with medication, eight of them (57.1%) converted to the nonfatigue group. Among the nonfatigue group at the follow-up, eleven patients (16.7%) converted to the fatigue group and 55 (83.3%) remained in the nonfatigue group (Figure 1(b)). Logistic regression analysis demonstrated that the changes of BDI and the presence of OH at the baseline were the predictors for fatigue in drug-naïve PD (Table 2).

## 4. Discussion

We investigated the progression of fatigue and factors associated with fatigue in drug-naïve PD and firstly studied the longitudinal association between fatigue and OH in drug-naïve PD. The prevalence of fatigue and the mean PFS score were similar after a mean follow-up duration of 23.3 months. The fatigue group had the higher prevalence of OH, worsened autonomic symptoms, depression, anxiety, and sleep disturbance compared with the nonfatigue group. The progression of fatigue was associated with the presence of OH and the change of depression in drug-naïve PD. In contrast, age, sex, disease duration, anxiety, and sleep disturbance were not significantly associated with the progression of fatigue.

Autonomic dysfunction of PD has been reported as an associated factor with fatigue in several cross-sectional studies [3, 10, 11], but none of the previous longitudinal studies focused on autonomic dysfunction [4–9]. The pathophysiology of fatigue in PD is largely unknown, and autonomic dysfunction is a suggested hypothesis that explains the pathophysiology of fatigue in PD [12]. The longitudinal association of the OH and fatigue suggested that the cumulative effect of OH or fluctuation of blood pressure might affect fatigue of the PD patients [10]. Several medications have been suggested for treating fatigue in PD, including dopaminergic medications, psychostimulants, doxepin, and tricyclic antidepressants, but the clinical usefulness is currently limited due to lack of compelling evidence [2, 12]. Based on the results of this study, managing OH can help moderate the extent and impact of fatigue, but additional evidence is needed for broader patient application.

Depression was also associated with the progression of fatigue. However, a longitudinal association between fatigue and depression showed inconsistent results in the previous studies. Siciliano et al. reported that depression was associated with the change of fatigue in a bivariate regression model but not in a hierarchical regression analysis [7]. Ongre and colleagues reported that depression measured by the Montgomery and Asberg Depression Rating Scale did not show a significant contribution of fatigue [6]. The discrepancies in the longitudinal studies can likely be attributable to the different regression analysis models among the studies. However, a systematic review and a meta-analysis study suggested that depression was a significantly associated factor with fatigue in PD. Our results are in agreement with the previous studies and suggest that OH and depression can play a pivotal role in the progression of fatigue in drug-naïve PD.

The prevalence of fatigue at the baseline in drug-naïve PD was 17.5%, and there was no change in the prevalence after the mean follow-up duration of 23.3 months. At the baseline of the study, the patients who reported fatigue were more likely to have OH, higher scores on the SCOPA-AUT, and have symptoms of depression (Supplementary table). This association is consistent with what we have found in our earlier study on fatigue in patients with drug-naïve PD [3]. Six of the 14 (42.9%) initially categorized patients in the fatigue group had persistent fatigue at the follow-up assessment. Only 11 of the initial nonfatigued patients converted to the fatigue group on the follow-up assessment. A systematic review study suggested that the prevalence of fatigue is similar across the levels of disease duration, which suggests that fatigue is present in the early phase of the disease and tends to persist over time [1]. Longitudinal studies have also shown that the overall prevalence of fatigue is similar over time, although these studies have also shown that, at the individual level, fatigue is not a persistent symptom but is changeable over time [4–7, 9]. Alves et al. reported that only 38.1% of PD patients had persistent fatigue, 24.2% had nonpersistent fatigue, and 25.7% did not experience fatigue during the 8-yearfollow-up [5]. Our results showed a similar result with the previous longitudinal studies that showed the changeability of fatigue. This result suggests that fatigue is not a persistent symptom, but other nonmotor symptoms including OH can affect the severity of fatigue in PD.

The fatigue group took higher levodopa dose and less MAO-B inhibitor compared to the nonfatigue group at the follow-up visit, but there was no difference in the LEDD. Although one previous randomized control study demonstrated that rasagiline improved fatigue in PD patients [25], there is no clear consensus about the benefit from PD medications on the fatigue in PD [26]. The fatigued patients tend to appeal more subjective symptoms than nonfatigued patients, and fatigued patients could have more change to initiate or increase the levodopa dose instead of using other antiparkinsonian medications. Therefore, the causality between medical treatment and fatigue should be interpreted with caution because of the small sample size and relatively short follow-up period in each group.

There were several limitations to this study. First, the follow-up duration differed from patient to patient, in contrast with the previous longitudinal studies that conducted a follow-up for 1 year [6, 7]. However, the duration in this study allowed the analysis to estimate the effect of disease duration on fatigue in drug-naïve PD patients, and the study demonstrated that the follow-up duration was not significantly associated with the progression of PFS. Additionally, OH can also be improved or newly developed with disease progression and antiparkinsonian medications, but there was no follow-up HUT for the present study. However, the study revealed that the presence of OH at the baseline has the predictive value for the progression of fatigue. To elucidate the details of the association between OH and fatigue in PD, follow-up data and analysis can be helpful.

## 5. Conclusion

In conclusion, fatigue is a common NMS with a prevalence of 17.5% in drug-naïve PD, and it is not persistent but can change during the disease course. OH and depression are the most relevant predictors for the worsening of fatigue in drug-naïve PD. Considering the diverse results of the previous studies, fatigue might result from multiple pathomechanisms, and the present study suggests that the two main pathomechanisms of fatigue in PD patients would be OH and depression. Based on our results, fatigue in PD should be assessed with a comprehensive approach to OH and depression for successful management in PD patients. Given that fatigue is a disabling symptom of PD, further studies are needed to confirm the results and best treatment approaches for patients.

## Figures and Tables

**Table 1:** Baseline and follow-up demographics and clinical characteristics of fatigue and nonfatigue groups.

	Baseline^a^			Follow-up^b^		
	Fatigue (*n* = 14)	Nonfatigue (*n* = 66)	*p* value	Fatigue (*n* = 17)	Nonfatigue (*n* = 63)	*p* value
Age (years)	66.6 ± 11.5	63.8 ± 8.4	0.301^c^	68.1 ± 12.7	67.9 ± 7.7	0.955^c^
Sex (M/F)	8/6	37/29	0.941^e^	7/10	38/25	0.158^e^
Disease duration (m)	20.4 ± 11.3	19.9 ± 11.9	0.875^c^	—	—	—
Follow-up duration (m)	—	—	—	27.2 ± 9.1	22.3 ± 9.9	0.070^c^
UPDRS III	19.7 ± 8.1	17.2 ± 8	0.344^c^	12.7 ± 6.1	14.7 ± 7.3	0.195^c^
H&Y stage	1.8 ± 0.5	1.6 ± 0.5	0.210^c^	1.7 ± 0.5	1.7 ± 0.5	0.785^c^
MoCA	25.4 ± 3.3	25.4 ± 3.1	0.977^c^	26.33 ± 2.5	26.3 ± 3.3	0.981^c^
PFS	3.6 ± 0.1	1.8 ± 0.7	<0.001^c^	3.8 ± 0.5	1.7 ± 0.7	<0.001^c^
OH *n* (%)	8 (57.1%)	17 (25.8%)	0.021^e^	—	—	—
SCOPA-AUT	19.3 ± 6.9	10.2 ± 5.5	<0.001^c^	19.8 ± 7.3	13 ± 8.5	0.003^c^
Gastrointestinal	4.3 ± 3.2	2.4 ± 2.4	0.036^d^	5.6 ± 3.1	3.2 ± 3.5	0.002^d^
Urinary	5.4 ± 3.9	3.4 ± 2.3	0.075^d^	4.8 ± 4.3	4.6 ± 4	0.934^c^
Cardiovascular	1.9 ± 3.0	0.4 ± 0.7	<0.001^d^	2.3 ± 2.2	0.5 ± 0.8	<0.001^d^
Thermoregulatory	2.1 ± 2.2	0.6 ± 1.0	0.002^d^	1.7 ± 2.2	0.7 ± 1.4	0.067^d^
Pupillomotor	0.4 ± 0.5	0.2 ± 0.4	0.211^d^	0.4 ± 0.7	0.2 ± 0.4	0.434^d^
Sexual	5.2 ± 3.4	3.3 ± 3.4	0.088^d^	5.6 ± 3.5	4.1 ± 3.4	0.257^d^
BDI	17.7 ± 10.3	7.1 ± 6.1	0.001^c^	14.6 ± 8.4	5.8 ± 5.9	<0.001^c^
BAI	11.5 ± 9.3	5.4 ± 4.7	0.023^c^	8.8 ± 7.1	5.0 ± 5.5	0.043^d^
PDSS	112.0 ± 19.9	126.3 ± 21.4	0.025^c^	109.2 ± 26.3	125.1 ± 23.8	0.019^c^
LEDD (mg)				363.3 ± 150	323.2 ± 179.3	0.401^c^
Levodopa (mg)	—	—	—	260.3 ± 134.9	158.7 ± 160.8	0.020^c^
Dopamine agonists (mg)	—	—	—	69.3 ± 52.4	88.7 ± 81.4	0.354^c^
COMT inhibitor				2 (11.8%)	3 (4.8%)	0.290^f^
MAO-B inhibitor	—	—	—	3 (17.6%)	32 (50.8%)	0.014^e^
Anticholinergics	—	—	—	1 (5.9%)	9 (14.2%)	0.353^e^
Antidepressants and/or anxiolytics	—	—	—	4 (23.5%)	7 (11.1%)	0.187^e^

UPDRS: united Parkinson's disease rating scale; H&Y: Hoehn & Yahr; TD: tremor dominant; AR: akinetic-rigid; MoCA: Montreal cognitive assessment; PFS: Parkinson fatigue scale; BDI: beck depression inventory; BAI: beck anxiety inventory; SCOPA-AUT: scale for outcomes of Parkinson's disease-autonomic; PDSS: Parkinson's disease sleep scale; LEDD: levodopa equivalent daily dose; COMT: catechol-O-methyltransferase; MAO-B: monoamine oxidase-B. ^a^assessments were performed before taking antiparkinsonian medications. ^b^assessments were performed during an on-state for PD medications. ^c^Student's *t*-test was used for statistical comparisons. ^d^the Mann–Whitney *U* test was used for statistical comparisons. ^e^the chi-square test was used for statistical comparisons. ^f^Fisher's exact test was used for statistical comparisons. Expressed as the number of patients (%), mean ± SD.

**Table 2:** Logistic regression analysis to predict figures in patients with Parkinson's disease.

Clinical characteristics (*n* = 80)	Estimate (SE)	Odds ratio (95% CI)	*p* value
Age (years)	0.013 (0.042)	1.013 (0.933, 1.101)	0.752
Sex (ref. = female)	0.886 (0.703)	2.425 (0.611, 9.620)	0.208
Disease duration (m)	0.049 (0.034)	1.051 (0.982, 1.124)	0.152
Follow-up duration (m)	0.080 (0.044)	1.084 (0.994, 1.182)	0.069
OH+	1.631 (0.754)	5.111 (1.167, 22.383)	0.030
SCOPA-AUT^a^	0.163 (0.388)	1.177 (0.550, 2.521)	0.674
BDI^a^	1.257 (0.550)	3.514 (1.197, 10.317)	0.022
BAI^a^	−0.251 (0.485)	0.778 (0.301, 2.014)	0.605
PDSS^a^	−0.775 (0.407)	0.461 (0.207, 1.024)	0.057

SE: standard error; CI: confidence interval; OH: orthostatic hypotension; SCOPA-AUT: scale for outcomes in Parkinson's disease-autonomic; BDI: beck depression inventory; BAI: beck anxiety inventory; PDSS: Parkinson's disease sleep scale. ^a^Standardized values were calculated by the following formula: [(mean follow-up score−mean baseline score)/baseline score SD].

## Data Availability

The data presented in this work are available from the corresponding authors upon request.

## Supplementary Materials

Supplementary Materials
*Supplementary Table.* Logistic regression analysis for fatigue and associated factors. Logistic linear regression analysis to identify the associated factors for the baseline fatigue in patients with clinical and demographic factors. We included factors that had a p value less than 0.1 in our univariate analysis (OH, the SCOPA-AUT, BDI, BAI, and PDSS) and clinically significant variables (age, sex, and disease duration). The results showed that OH, SCOPA-AUT, and BDI were the associated factors with baseline fatigue.
