# Supplementary material for: Orthostatic Hypotension Is a Predictor of Fatigue in Drug-Naïve Parkinson's Disease
Source: Parkinsons Dis. 2023 Feb 8;2023:1700893. doi: 10.1155/2023/1700893 (PMC9931477; doi:10.1155/2023/1700893)
Supplement: Supplementary Materials — Supplementary Table. Logistic regression analysis for fatigue and associated factors. Logistic linear regression analysis to identify the associated factors for the baseline fatigue in patients with clinical and demographic factors. We included factors that had a p value less than 0.1 in our univariate analysis (OH, the SCOPA-AUT, BDI, BAI, and PDSS) and clinically significant variables (age, sex, and disease duration). The results showed that OH, SCOPA-AUT, and BDI were the associated factors with baseline fatigue. [file 1700893.f1.docx]

Supplementary Table . Logistic regression analysis for fatigue and associated factors.

| Clinical characteristics (*n* = 80) | Estimate (SE) | Odds Ratio (95% CI) | *p* value |
| --- | --- | --- | --- |
| Age (years) | 0.007 (0.049) | 1.007 (0.914, 1.110) | 0.883 |
| Sex (ref. = female) | -0.710 (1.159) | 0.492 (0.051, 4.764) | 0.540 |
| Disease duration (m) | -0.038 (0.046) | 0.963 (0.881, 1.053) | 0.405 |
| OH+ | 2.722 (1.152) | 15.207 (1.59, 145.456) | 0.018 |
| SCOPA-AUT | 0.341 (0.123) | 1.406 (1.105, 1.790) | 0.006 |
| BDI | 0.331 (0.143) | 1.392 (1.051, 1.843) | 0.021 |
| BAI | -0.175 (0.128) | 0.840 (0.654, 1.078) | 0.171 |
| PDSS | 0.06 (0.041) | 1.062 (0.980, 1.151) | 0.142 |

SE, standard error; CI, Confidence Interval; UPDRS; OH, Orthostatic Hypotension; SCOPA-AUT, Scale for Outcomes in Parkinson’s Disease-Autonomic; BDI, Beck Depression Inventory; PDSS, Parkinson’s Disease Sleep Scale.

**Statistical Analysis**

Logistic linear regression analysis to identify the associated factors for the baseline fatigue in patients with clinical and demographic factors. We included factors that had a p value less than 0.1 in our univariate analysis, such as orthostatic hypotension, the SCOPA-AUT, BDI, BAI, and PDSS, as well as factors that have been found to be clinically significant in previous studies, such as age, sex, and disease duration.
